# Supplementary material for: Complexome profiling of the Chlamydomonas psb28 mutant reveals TEF5 as an early PSII assembly factor
Source: Plant Cell. 2025 Mar 17;37(6):koaf055. doi: 10.1093/plcell/koaf055 (PMC12164590; doi:10.1093/plcell/koaf055)
Supplement: koaf055_Supplementary_Data [file koaf055_supplementary_data.zip › psb28_interactive.zip!/plots/intensity/qualityPlots/peptides/HCF136_Cre06.g273700.t1.2.html]

### Cre06.g273700.t1.2

Trivial name: HCF136  
SeqCoverage [median]: 48.60  
Mass: 43.16 kDa  
  
 

| Sequence | MissedCleavage | Mass [Da] | wtIntensity | muIntensity | Score |
| --- | --- | --- | --- | --- | --- |
| DEGFNYR | 0 | 899.38 | 10134.16 | 50356.32 | 162.51 |
| DRSTDDVAGNLYAIK | 1 | 1636.81 | 9147.78 | 53610.23 | 121.99 |
| EGWIVGKPAILLHTSDGGANWER | 1 | 2505.28 | 0.00 | 2070.30 | 42.90 |
| FKDVTINSR | 1 | 1078.58 | 25640.21 | 118024.41 | 121.61 |
| FNSISFAGK | 0 | 969.49 | 22162.97 | 114213.14 | 134.48 |
| GFGILDVGFR | 0 | 1079.58 | 1370.77 | 0.00 | 82.45 |
| GFGILDVGFRDDK | 1 | 1437.73 | 473.56 | 12979.27 | 84.29 |
| GGDVLVSEDAGLASEK | 0 | 1545.75 | 34241.41 | 159045.95 | 286.19 |
| GGDVLVSEDAGLASEKFK | 1 | 1820.92 | 0.00 | 1508.19 | 48.31 |
| GGDVLVSEDAGLASEKFKDVTINSR | 2 | 2606.32 | 0.00 | 13357.61 | 64.49 |
| HGFLLGTR | 0 | 899.50 | 0.00 | 2512.67 | 107.59 |
| IPLSSKLPGAPVR | 1 | 1333.81 | 5330.71 | 25325.20 | 113.24 |
| LPGAPVR | 0 | 708.43 | 38937.69 | 145783.04 | 163.54 |
| LQNMGFTPAGEVWVTTR | 0 | 1905.94 | 0.00 | 5117.44 | 101.62 |
| PAILLHTSDGGANWER | 0 | 1735.86 | 0.00 | 4409.71 | 83.69 |
| QTLLETNDGGK | 0 | 1174.58 | 10811.35 | 57081.62 | 238.86 |
| QTLLETNDGGKTWAPR | 1 | 1785.90 | 963.74 | 7722.08 | 97.29 |
| RLQNMGFTPAGEVWVTTR | 1 | 2062.04 | 9190.76 | 67570.28 | 153.36 |
| SEDGGKNWKR | 2 | 1175.57 | 407.68 | 6686.52 | 113.93 |
| SIDAARDEGFNYR | 1 | 1512.70 | 18182.28 | 72835.29 | 110.29 |
| SSSGDYVAVSSR | 0 | 1213.56 | 41015.84 | 171653.04 | 225.22 |
| STDDVAGNLYAIK | 0 | 1365.68 | 30975.46 | 185992.63 | 300.50 |
| TVSSGISGASYYEGSFSNLAR | 0 | 2152.01 | 8536.20 | 25402.21 | 204.18 |
|  |  |  | 267522.6 | 1303257.1 |  |
